# Microporous Dermal-Mimetic Electrospun Scaffolds Pre-Seeded with Fibroblasts Promote Tissue Regeneration in Full-Thickness Skin Wounds

**DOI:** 10.1371/journal.pone.0122359

**Published:** 2015-03-20

**Authors:** Paul P. Bonvallet, Matthew J. Schultz, Elizabeth H. Mitchell, Jennifer L. Bain, Bonnie K. Culpepper, Steven J. Thomas, Susan L. Bellis

**Affiliations:** 1 Department of Cell, Developmental and Integrative Biology, University of Alabama at Birmingham, Birmingham, Alabama, United States of America; 2 Department of Periodontology, University of Alabama at Birmingham, Birmingham, Alabama, United States of America; 3 Department of Biomedical Engineering, University of Alabama at Birmingham, Birmingham, Alabama, United States of America; 4 Department of Surgery, University of Alabama at Birmingham, Birmingham, Alabama, United States of America; University of California, San Diego, UNITED STATES

## Abstract

Electrospun scaffolds serve as promising substrates for tissue repair due to their nanofibrous architecture and amenability to tailoring of chemical composition. In this study, the regenerative potential of a microporous electrospun scaffold pre-seeded with dermal fibroblasts was evaluated. Previously we reported that a 70% collagen I and 30% poly(Ɛ-caprolactone) electrospun scaffold (70:30 col/PCL) containing 160 μm diameter pores had favorable mechanical properties, supported fibroblast infiltration and subsequent cell-mediated deposition of extracellular matrix (ECM), and promoted more rapid and effective *in vivo* skin regeneration when compared to scaffolds lacking micropores. In the current study we tested the hypothesis that the efficacy of the 70:30 col/PCL microporous scaffolds could be further enhanced by seeding scaffolds with dermal fibroblasts prior to implantation into skin wounds. To address this hypothesis, a Fischer 344 (F344) rat syngeneic model was employed. *In vitro* studies showed that dermal fibroblasts isolated from F344 rat skin were able to adhere and proliferate on 70:30 col/PCL microporous scaffolds, and the cells also filled the 160 μm pores with native ECM proteins such as collagen I and fibronectin. Additionally, scaffolds seeded with F344 fibroblasts exhibited a low rate of contraction (~14%) over a 21 day time frame. To assess regenerative potential, scaffolds with or without seeded F344 dermal fibroblasts were implanted into full thickness, critical size defects created in F344 hosts. Specifically, we compared: microporous scaffolds containing fibroblasts seeded for 4 days; scaffolds containing fibroblasts seeded for only 1 day; acellular microporous scaffolds; and a sham wound (no scaffold). Scaffolds containing fibroblasts seeded for 4 days had the best response of all treatment groups with respect to accelerated wound healing, a more normal-appearing dermal matrix structure, and hair follicle regeneration. Collectively these results suggest that microporous electrospun scaffolds pre-seeded with fibroblasts promote greater wound-healing than acellular scaffolds.

## Introduction

Skin tissue performs numerous functions such as defense against invading pathogens, protection from physical insults, storage of water and lipids, and touch and pain sensation. The gold standard therapy for severely damaged skin is autografting; however, this is only an option if the patient has sufficient unwounded skin tissue for transplantation. The limited amount of available donor autograft tissue, secondary wound site creation, and uneven appearance of the regenerated skin due to meshing of the donor tissue are undesirable features of autografting, prompting the need for alternative approaches. Alternative therapies include allografts and xenografts, but these also have limitations such as graft contraction, weak mechanical properties, rejection, and scar formation [1–4]. For these reasons, numerous groups are engineering graft materials that can substitute for current therapies [5,6].

Engineered scaffolds typically consist of synthetic polymers such as poly (ε-caprolactone) (PCL) or Poly(3-hydroxybutyrate-co-3-hydroxyvalerate) (PHBV), natural biochemical compounds,or a combination of these [7–16]. Synthetic polymers are used in graft materials because they are FDA approved, biodegradable, and have favorable mechanical characteristics [17]. Natural extracellular matrix (ECM)-derived materials such as collagen, hyaluronan, and elastin are used because they promote cell attachment and survival, and mimic the microenvironment native to human skin [18,19]. However, scaffolds derived from natural ECM molecules often have low mechanical strength and fast degradation rates. Therefore, many groups combine natural and synthetic materials to create scaffolds that have cell instructive biochemical elements as well as suitable mechanical properties. Furthermore, the incorporation of biologics other than ECM, such as growth or angiogenic factors, represents a major area of research interest [20–23]. While many technologies for combining biologic and synthetic components into scaffolds are currently being investigated, electrospinning offers a promising approach. Electrospun scaffolds have a high surface to volume ratio, which promotes cell adhesion, interconnected pores that facilitate nutrient transport and waste removal, and nanofibers that resemble native ECM [24,25].

For skin regeneration, electrospun materials have one major shortfall; nanopores spanning the scaffold are typically too small to allow efficient fibroblast migration throughout the entirety of the scaffold [26]. Many groups are investigating ways to increase scaffold pore size by using methods such as inclusion of sacrificial particles or fibers, or through changes in the electrospinning apparatus and/or protocol [27–31]. While some of these approaches have been successful, disadvantages include the difficulty in achieving reproducible pore size and distribution, the need for complicated or expensive experimental set-ups, and the possibility of residual cytotoxic material from sacrificial elements. To address this issue, our group has investigated a cost-effective and simple approach for increasing scaffold pore size [32]. Specifically, micropores are created mechanically in electrospun scaffolds using needles with a micron-scale diameter. This method generates pores of well-defined size and shape, and can be applied to any type of electrospun formulation.

Our prior studies focused on developing a skin regenerative scaffold with optimal biochemical composition, mechanical properties, degradation kinetics, and pore diameter for cell infiltration. We examined multiple scaffold compositions and determined that a combination of 70% collagen I and 30% PCL (70:30 col/PCL) yielded a substrate that supported dermal fibroblast attachment and proliferation, while still maintaining appropriate mechanical properties for skin tissue regeneration [32]. Additionally, it was found that the introduction of 160 μm pores into the 70:30 col/PCL scaffolds enhanced fibroblast infiltration, as well as fibroblast-mediated filling of the micropores with native ECM molecules. To evaluate regenerative potential of the 70:30 col/PCL scaffolds with 160 μm pores, scaffolds were implanted into full-thickness skin defects. When compared with scaffolds lacking micropores or sham wounds, the 70:30 col/PCL scaffolds with 160 μm pores expedited the wound healing process, assisted in re-epithelialization and follicle regeneration, and promoted the formation of dermal tissue with a matrix architecture resembling normal, unwounded skin.

Recent studies have highlighted the importance of pre-seeding skin or stem cells on a scaffold prior to implantation in order to, “jump start,” the ECM remodeling process and rate of implant integration [33–38]. The goal of the current study was to determine whether 70:30 col/PCL microporous scaffolds with pre-seeded dermal fibroblasts have a greater regenerative capacity than acellular microporous scaffolds. A syngeneic Fischer 344 (F344) rat model was used to evaluate the performance of fibroblast-seeded scaffolds. We first conducted *in vitro* studies to confirm that, as with human dermal fibroblasts, F344 fibroblasts proliferated on 70:30 col/PCL scaffolds with 160 μm pores, and filled the micropores with ECM. Subsequently, scaffolds pre-seeded with F344 fibroblasts for either 1 or 4 days, or alternatively, acellular microporous scaffolds, were implanted into full-thickness critical size skin defects created in F344 hosts. It was found that scaffolds pre-seeded with fibroblasts for 4 days stimulated the greatest degree of skin regeneration, although both of the fibroblast-seeded scaffolds promoted better skin healing than acellular scaffolds or sham wounds.

## Materials and Methods

### Preparation of electrospun scaffolds

**Fig 1:**
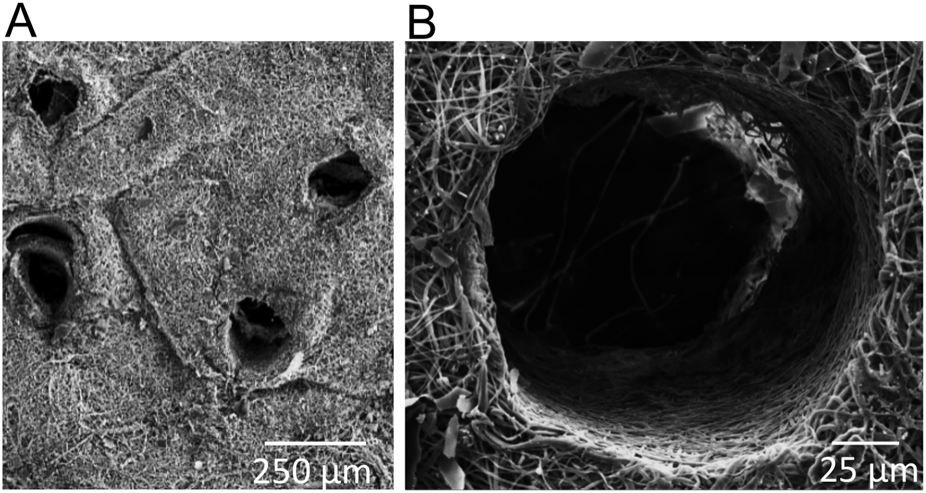
Scanning electron microscopy (SEM) images of scaffolds with 160 μm pores. A) Magnification of 150x. B) Magnification of 1834x.

Hexafluoroisopropanol (HFP) (Sigma), an organic solvent, was used to dissolve 70% collagen I and 30% PCL into solution. The collagen I was derived from calf skin (MP Biomedicals) and the 10,000 Da PCL was purchased from Scientific Polymer Products. After the solution was taken up into a 3-cc syringe with a 27-gauge needle, it was ejected at a constant 2 mL/h rate by the use of a syringe pump (Harvard Apparatus). The needle was charged with 20 KV (Gamma High Voltage Research) so that when the solution was ejected, it naturally traveled 20 cm horizontally to a grounded collecting plate. The collecting plate was covered in a thin aluminum sheet and rotated at 20 rpm for even fiber distribution. Scaffolds were then placed in a desiccator for 24 h to remove any residual HFP. A Humboldt Boring Machine (Fisher) was used to punch circular scaffolds with a 15 mm diameter. Micropores were created mechanically using 160 μm acupuncture needles as described in [32]. For each 15 mm diameter scaffold, 150 pores were created throughout in order to maintain a constant number across all experiments. To validate pore size and distribution, scanning electron microscopy (SEM) images were taken (Fig. 1). Briefly, scaffolds were dried in a desiccator for 24 h, perforated with 160 μm diameter needles, gold plated, and imaged using a FEI FEG 650 SEM with an accelerating voltage of 10 kV in SE mode. At least 40 pores were imaged and measured to establish that the average pore diameter ranged from 150–175 μm, and the average spacing between pores was 375 μm.

### Fischer 344 cell viability and proliferation on scaffolds

A syngeneic Fischer 344 (F344) rat model was employed in order to avoid potential rejection of implanted cells in *in vivo* studies. Dermal fibroblasts were isolated from F344 rat skin by the Skin Cell Culture Core Facility at the University of Alabama at Birmingham (UAB). F344 cells were extracted from skin tissue by standard methods [39] and cultured in Dulbecco’s modified Eagle’s medium that was supplemented with 10% fetal bovine serum (FBS) and 1% penicillin/streptomycin/amphotericin solution (Invitrogen). To avoid potential phenotypic alteration in culture, all studies were performed with fibroblasts from passages 3–5.

F344 fibroblasts were seeded on 15 mm diameter scaffolds at a density of 35,000 and allowed to grow for varying time intervals between 1 and 14 days. At each time point, the scaffolds were submerged in a live/dead cell imaging solution (Life Technologies) for 15 min. Live cells were stained green and dead cells red. The scaffolds were then rinsed in phosphate-buffered saline (PBS) and imaged on a Nikon confocal microscope. In order to determine the number of live and dead cells, the Volocity image analysis software program was used for cell quantification.

### Scaffold contraction

F344 fibroblasts were seeded onto microporous electrospun scaffolds at a density of 35,000 cells per 15 mm diameter scaffold and grown in culture for up to 21 days. At each time point, the diameters of 5 separate scaffolds per group were measured with calipers (Fisher). The initial diameter was also measured so that the difference in diameters could be determined, and the percent contraction calculated.

### Fibroblast-mediated ECM deposition into scaffold pores

F344 fibroblasts (35,000 cells) were seeded onto 15 mm diameter microporous scaffolds and allowed to grow for up to 14 days. At each time point, deposition of a fibrous matrix within the micropores was visualized using a phase-contrast dissecting microscope. To monitor cell infiltration into the scaffold, fibroblasts were pre-labeled with red nanocrystals (Invitrogen) and then seeded onto scaffolds with or without introduced micropores. At 10 days after initial seeding, scaffolds were cross-sectioned, stained with Hoechst, and imaged. The deposition of fibrous collagen within micropores was assessed by embedding scaffolds in Optimal Cutting Temperature (OCT) substrate and then staining with picrosirius red. OCT blocks were created by placing scaffolds vertically in OCT gel, followed by freezing in liquid nitrogen, and storage at -20°C until use. OCT-embedded scaffolds were then vertically cross-sectioned, fixed in 4% paraformaldehyde, and stained using a picrosirius red stain kit (Polysciences, Inc.). The deposition of fibronectin and collagen I within micropores was evaluated by immunoblotting. For each time point, five separate scaffolds were combined together, rinsed in PBS, frozen in liquid nitrogen, and pulverized using a cryo-pulverizer. A 50 mM Tris buffer (pH 7.4) lysis solution containing 150 mM NaCl, 1% Triton X-100, 1% deoxycholate, 0.1% SDS, 5 mM EDTA, and 0.5% Igepal was used to lyse cells in scaffolds. Scaffold homogenates were centrifuged at 15,000 g for 20 min, and supernatants collected. Samples were resolved by SDS-PAGE, and transferred to a polyvinylidene fluoride (PVDF) membrane. Membranes were blocked in 5% milk, incubated with primary antibodies against fibronectin or collagen I (Abcam) and then with secondary antibody. A BioRad ChemiDoc imaging system was used for imaging.

### Scaffold implantation into full-thickness skin defects

All animal procedures were performed with approval from the Institutional Animal Care and Use Committee (IACUC) at UAB. Four 15-mm-diameter full-thickness skin defects were created side-by-side in the back skin of F344 rats (*n* = 5). One of the wounds was implanted with a 70:30 col/PCL microporous scaffold lacking any cells; a second defect was implanted with a 70:30 col/PCL microporous scaffold seeded with 35,000 fibroblasts for 4 days, and a third defect was implanted with a 70:30 col/PCL microporous scaffold seeded with 35,000 fibroblasts for 1 day. Scaffolds were sutured into place. The fourth wound was covered with gauze only (no scaffold) to serve as a sham control. At 7, 14, and 21 days, top-down images of the wound surface were taken, and then the scaffolds and surrounding tissues were harvested. Samples were paraffin embedded, sectioned, and H&E stained. Whole field, as well as high magnification, images of the wound bed were taken and the abnormal tissue area, as well as the basket-weave matrix resembling native skin tissue, were measured with Image J analysis software.

## Results

### Viability of fibroblasts grown on scaffolds

**Fig 2:**
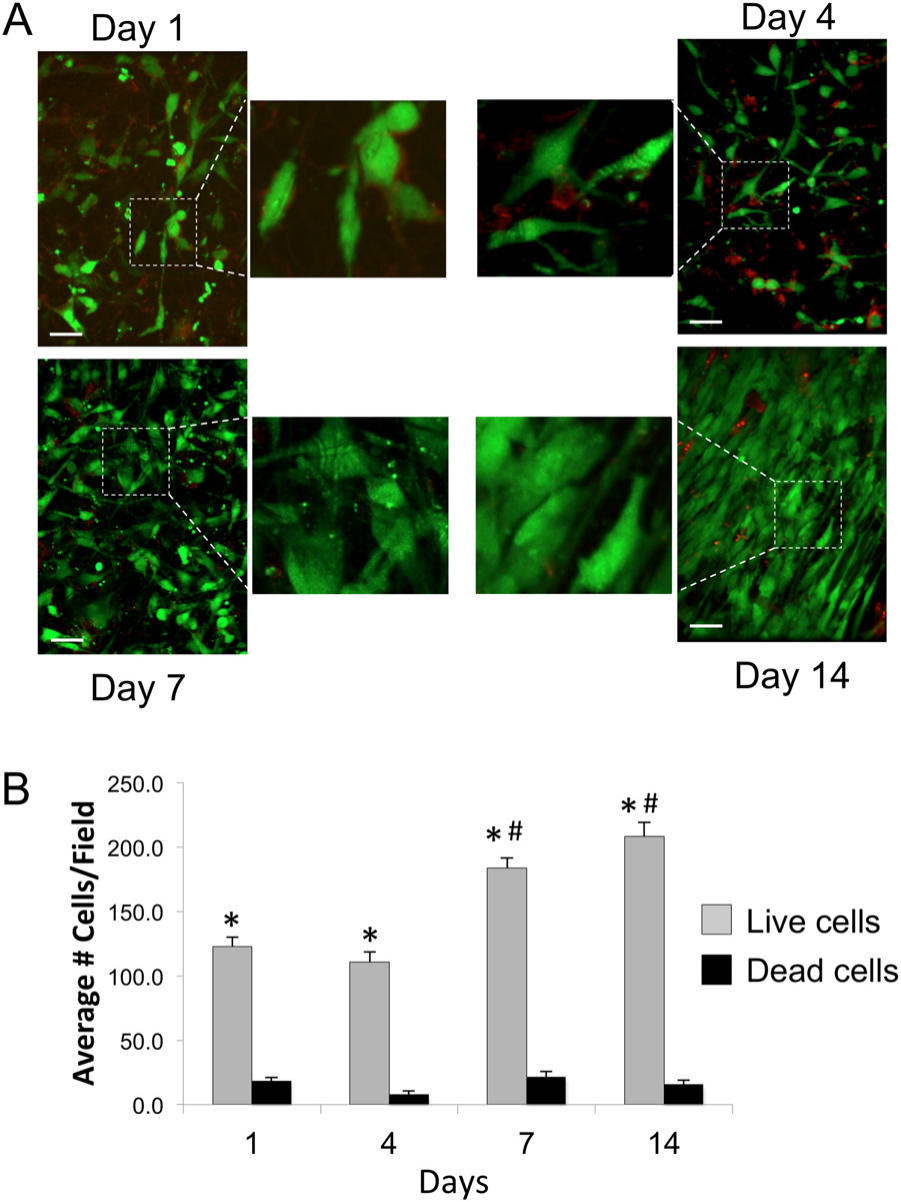
Fibroblast viability on 70:30 col/PCL scaffolds. **(A)** F344 fibroblasts grown on scaffolds for 1, 4, 7, and 14 days were stained for either living (green) or dead (red) cells. Scale bar = 40 μm. **(B)** Values represent means and standard error of the mean for live and dead cells measured from three distinct fields per scaffold, with multiple scaffolds evaluated. * represents significant difference in live cell number compared to dead cell number at each time point (p<0.05); # represents difference in live cell number relative to live cell number at day 1 (p<0.05).

To determine whether cells were able to adhere to, and survive on, the scaffolds, we performed live/dead cell staining on scaffolds cultured with F344 fibroblasts for 1, 4, 7, or 14 days. As shown in Fig. 2A, the number of viable cells appeared to increase over the 14 day interval, consistent with cell proliferation, and by 14 days the cells were confluent. Quantification of the staining (Fig. 2B) using the Volocity imaging software program confirmed an increase in viable cell number from 1 to 14 days, and also indicated that cell death was minimal.

### Fibroblast-seeded scaffolds have low contraction rates

**Fig 3:**
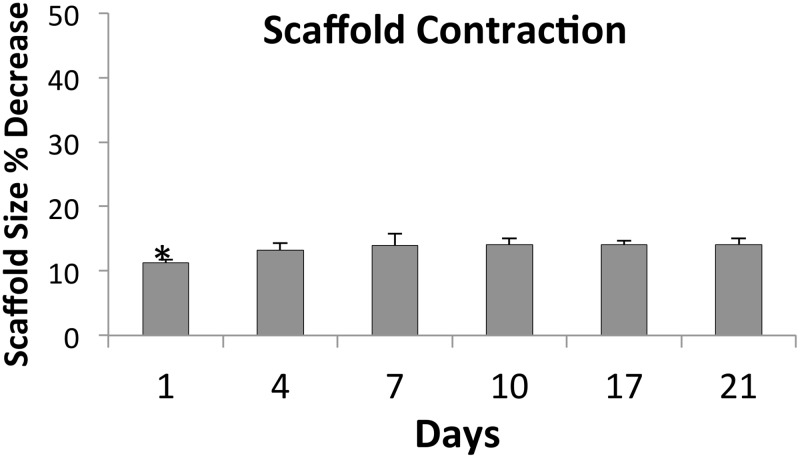
Contraction of porous 70:30 col/PCL scaffolds containing seeded fibroblasts. Scaffold diameters were measured at each time interval to quantify contraction (plotted as percent decrease in scaffold diameter). Values represent means and standard deviation for five scaffolds per time point. Two independent experiments were performed. A one way Anova was performed to compare the percent change in scaffold contraction of the various groups. *Represents significant difference (p<0.01) relative to all other groups.

Contraction is normal in the wound healing process; however, excessive contraction can cause pain, scarring and immobility [40]. Accordingly, we examined the degree of contraction exhibited by microporous scaffolds seeded with F344 fibroblasts for varying time intervals (Fig. 3). We observed an 11.3% decrease in scaffold size at 24 hours after seeding with fibroblasts. By 4 days, the amount of scaffold contraction leveled off at around 14%. These *in vitro* contraction rates cannot be directly compared to scaffold contraction within the wound bed; however, a value of 14% seems acceptable, given that many current commercial products can have contraction rates up to 50% [41,42].

### Infiltrating fibroblasts deposit ECM into scaffold micropores

**Fig 4:**
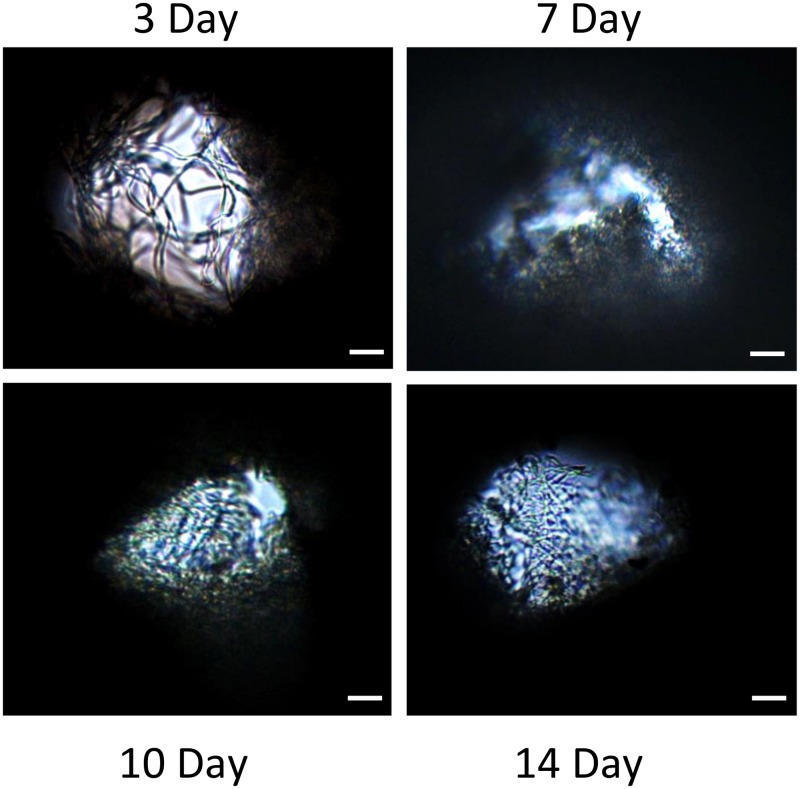
Extracellular matrix is deposited into the 160 μm pores of 70:30 col/PCL scaffolds. Top-down phase-contrast images of pores at 3, 7, 10, and 14 days following cell seeding reveal fibrous matrix deposition over time. Scale bar = 40 μm.

F344 fibroblasts were seeded onto microporous scaffolds and allowed to grow and secrete matrix for up to 14 days. At 3, 7, 10, and 14 days post cell-seeding, 20X top view images were taken to assess fibrous matrix deposition. It is apparent from the images in Fig. 4 that ECM deposition occurred gradually, with a small amount of pore filling observed within 3 days, and complete pore filling by 14 days.

**Fig 5:**
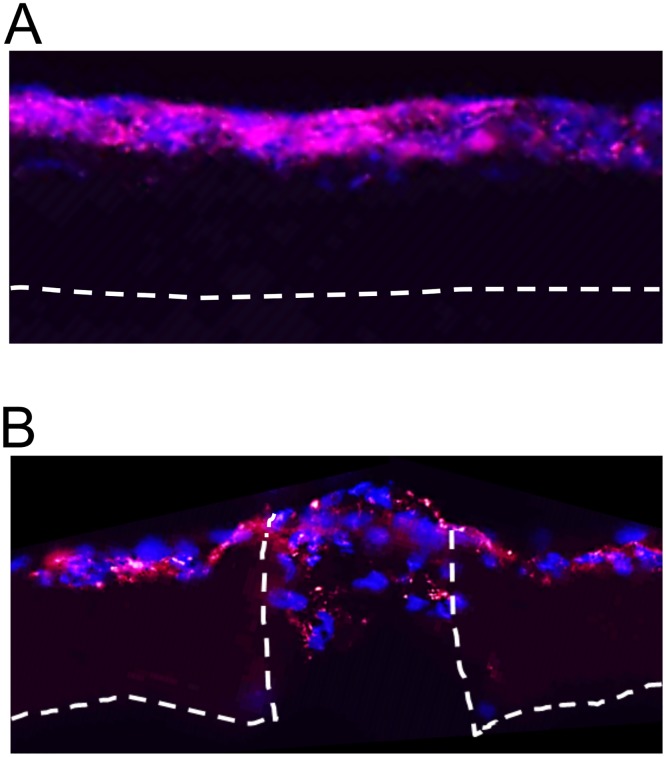
Fibroblast infiltration into scaffolds with or without 160 μm pores. Fibroblasts were pre-loaded with red nanocrystals and then seeded onto: **(A)** standard electrospun 70:30 col/PCL scaffolds (lacking micropores), or **(B)** 70:30 col/PCL scaffolds with introduced 160 μm micropores. After 10 days of cell culture, scaffolds were OCT-embedded, cross-sectioned, labeled with Hoescht, and imaged. Fibroblasts can be seen migrating into the micropores.

We next assessed fibroblast infiltration into either the microporous scaffolds, or standard electrospun scaffolds lacking micropores (Fig. 5). After 10 days of culture on the scaffolds lacking micropores (Fig. 5A), fibroblasts are found almost exclusively on the scaffold surface with minimal infiltration. In contrast, fibroblasts grown on scaffolds with 160 μm pores (Fig. 5B) clearly migrate deep within the micropores, fostering the deposition of matrix within these spaces.

**Fig 6:**
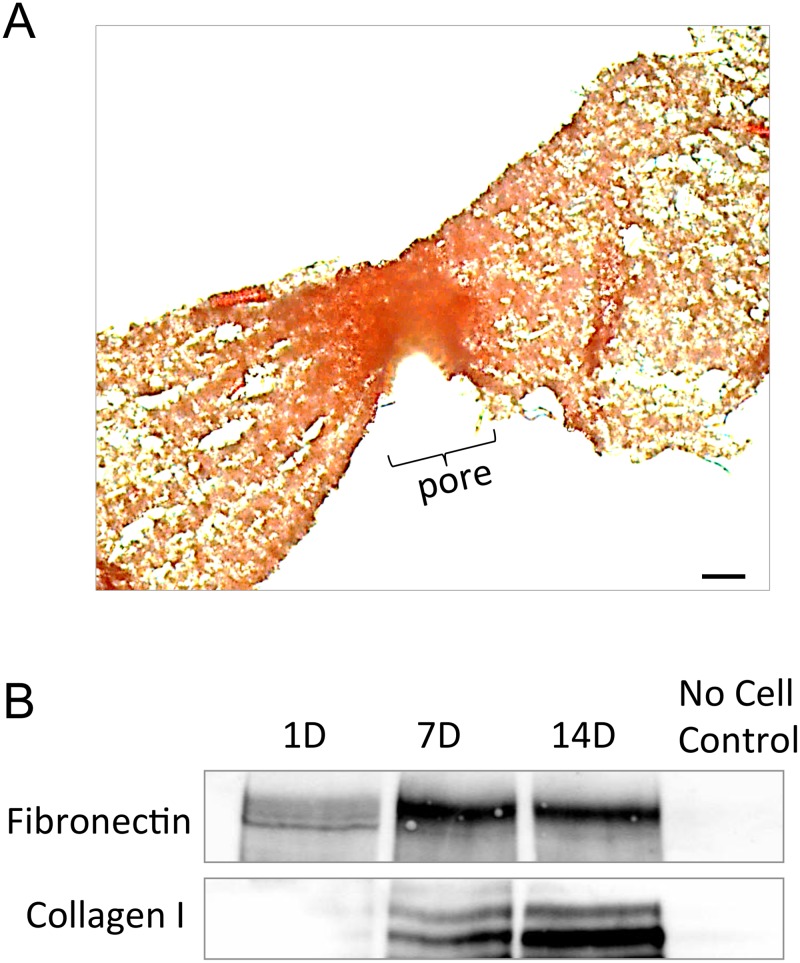
Collagen and fibronectin are deposited into 160 μm pores within scaffolds. **(A)** A picrosirius red stain was used to detect fibrillar collagen in 70:30 col/PCL scaffolds. Scale bar = 40 μm. **(B)** Immunoblotting for collagen I and fibronectin was performed on homogenates prepared from microporous scaffolds with adherent fibroblasts grown for 1, 7, and 14 days. The no cell negative control was prepared from scaffolds that were not seeded with fibroblasts.

To characterize the composition of cell-secreted matrix, scaffolds were stained with picrosirius red to detect fibrillar forms of collagen, which play an important role in wound healing. As shown in Fig. 6A, picrosirius red staining was enriched within the region of the micropore, suggesting that fibroblasts were filling the pores with collagen fibers (note that the electropun fibers have some staining due to the blended collagen/PCL composition). We also evaluated the deposition of fibronectin within scaffolds, as fibronectin is one of the critical ECM molecules within provisional wound healing matrices. To assess fibronectin deposition, scaffolds seeded with fibroblasts for varying time intervals were homogenized and fibronectin was detected by immunoblotting (Fig. 6B). The amount of fibronectin appeared to increase over time. Immunoblotting was similarly performed for collagen I, and it was found that collagen I deposition by fibroblasts also accumulated from day 1 to day 14.

### Scaffolds pre-seeded with fibroblasts facilitate regeneration of more normal appearing skin tissue than acellular scaffolds

Having shown that the scaffolds provided good support for fibroblast growth and ECM deposition, the next objective was to assess the capacity of scaffolds to promote wound healing when implanted into full-thickness critical size skin defects. We hypothesized that fibroblast-containing scaffolds with matrix-filled pores would enhance wound healing when compared with acellular microporous scaffolds. Given that maximal pore filling was observed by 10–14 days after cell seeding, our initial plan was to implant scaffolds cultured with fibroblasts for 10 days. However, after 10 days of culture, the scaffold handling properties had diminished greatly, and the scaffolds were too fragile to suture into place. Therefore, we adjusted our seeding time points to 1 and 4 days. Notably, some degree of matrix deposition is observed within pores by 3 days after seeding (Fig. 4).

**Fig 7:**
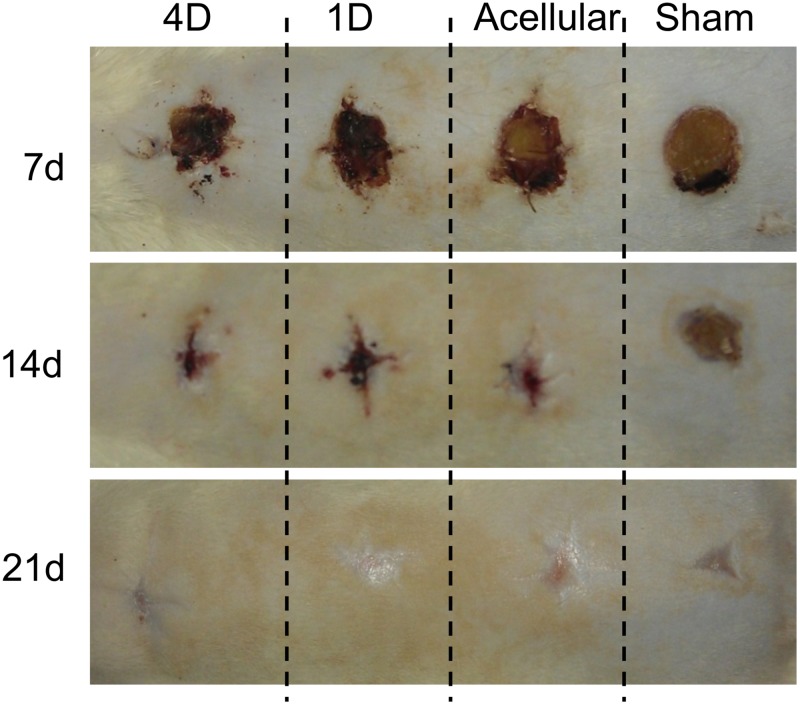
Images of skin wounds implanted with cell-seeded or acellular microporous scaffolds over a 21-day interval. Four full-thickness defects were created in the backskin of each rat, with 5 rats examined per time point. One wound was implanted with microporous scaffolds pre-seeded with fibroblasts for 4 days (4D), another was implanted with microporous scaffolds pre-seeded with fibroblasts for 1 day (1D), a third was implanted with an acellular microporous scaffold (acellular), and the final wound was covered with gauze only (no implant, “Sham”). Representative images are shown for each time point.

**Fig 8:**
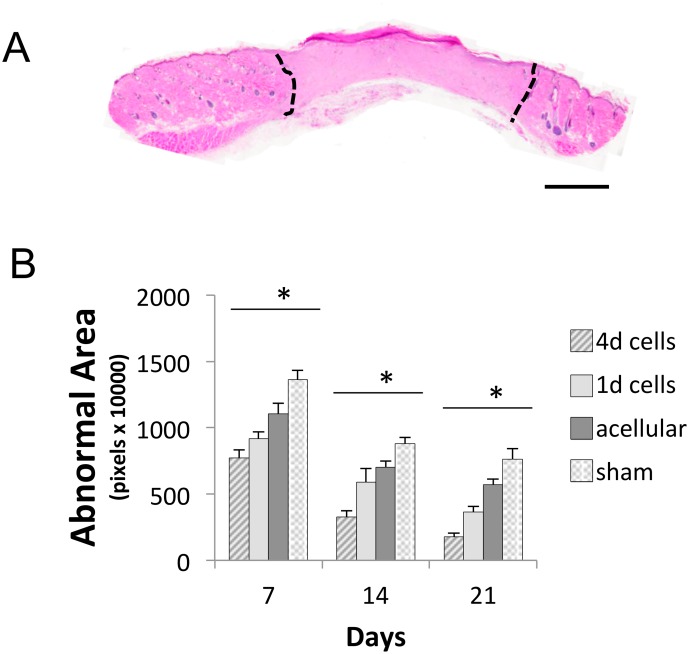
Pre-seeded scaffolds promote more effective tissue regeneration. **(A)** A cross-section of rat skin tissue undergoing the wound-healing process. Black dashed lines designate junctions between abnormal tissue and normal skin morphology. Scale bar = 40 μm. **(B)** Graph depicts average abnormal tissue areas (*n* = 5 rats per group) of harvested tissues containing porous scaffolds seeded for 4 days with fibroblasts (4d), porous scaffolds seeded for 1 day (1d), acellular porous scaffolds, and sham wounds. A repeated measure Anova was performed to compare significance between treatment groups. *Represents p<0.01 for all treatment groups that are significantly different from each other within the same time point. Also, all treatment groups significantly decreased over time (p<0.001).

Scaffolds cultured with F344 fibroblasts for 1 or 4 days were implanted into 15 mm-diameter full thickness defects created in the backskin of F344 rats. Microporous scaffolds lacking seeded fibroblasts were also placed into defects to assess the importance of cellular factors, including secreted ECM, in the wound healing response. Additionally, we created sham wounds (no scaffold) as a control. Wounds were allowed to heal for 7, 14 or 21 days (Fig. 7). No major differences were noted in the rate of superficial wound closure. To assess healing of the underlying dermal tissue, the scaffolds and surrounding tissues were harvested, paraffin embedded, sectioned, and stained with H&E. A dense matrix was observed within the wound area of many of the samples, consistent with scar-like tissue formation. To quantify the amount of this tissue, the junction between the abnormal tissue and the normal-appearing skin (with associated skin appendages) was designated with black dotted lines (Fig. 8A), and the relative area of abnormal tissue was quantified using Image J software. These studies (Fig. 8B) showed that scaffolds seeded with cells for 4 days prior to implantation elicited a smaller amount of abnormal tissue than all other treatment groups, at all three time points. It was also apparent that both of the scaffolds seeded with cells evoked less abnormal tissue formation than microporous scaffolds without pre-seeded cells. Sham wounds had the highest amount of abnormal tissue at all time points.

**Fig 9:**
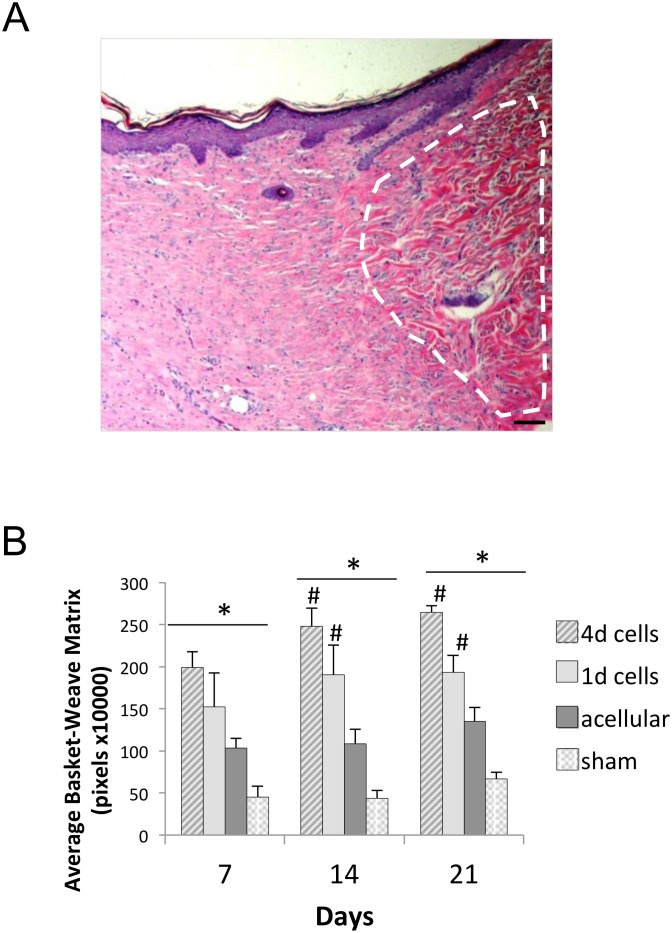
Scaffolds pre-seeded with fibroblasts promote formation of ECM with a high degree of basket-weave structure, resembling unwounded skin tissue. **(A)** Representative image of a cross-section of a wound bed harvested from a rat implanted with a cell-seeded microporous scaffold. White dashed line designates the area of basket-weave matrix. Scale bar = 40 μm. **(B)** Graph depicts the average basket-weave area of harvested tissues containing scaffolds seeded for 4 days with fibroblasts (4d), scaffolds seeded for 1 day (1d), acellular porous scaffolds, and sham wounds (*n* = 5 rats per group, with multiple microscopic fields examined per specimen). A repeated measure Anova was performed to compare significance between treatment groups. *Represents p<0.01 for all treatment groups that are significantly different from each other within the same time point. ^#^ Represents treatment groups significantly different than their respective groups at the 7 day time point (p<0.05).

**Fig 10:**
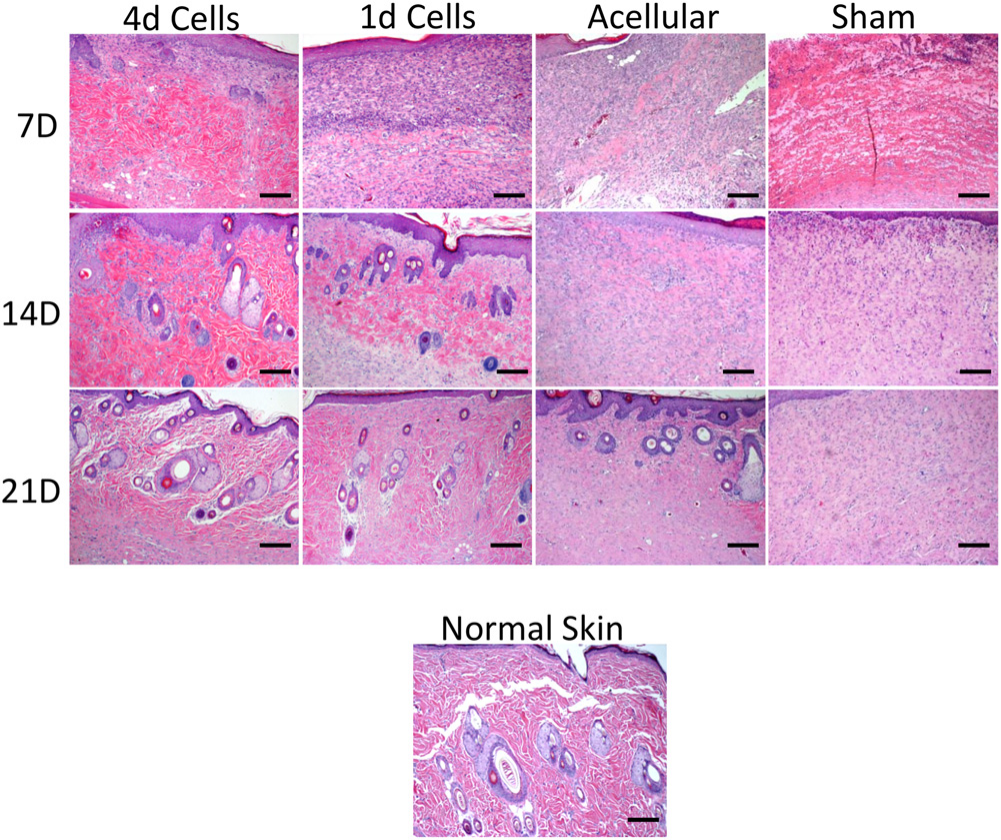
Images (10X) of wound healing over a 21-day time period show the structure of the matrix in each treatment group. The matrix in wounds containing scaffolds pre-seeded with fibroblasts appears more normal to skin tissue with loose, wavy basket-weave matrix and the formation of hair follicles. Scale bar = 10 μm.

Another notable feature observed in higher magnification images of the regenerated tissue was the architecture of the matrix. The dermal matrix of normal skin has a basket weave structure, represented by a loose wavy appearance [43]. This structure was observed in many of the samples. The area of matrix with a basket weave appearance, relative to areas of dense matrix suggestive of scar tissue, was measured as indicated in Fig. 9A. These studies suggested that the amount of basket-weave matrix was greatest in the wounds containing scaffolds seeded with fibroblasts for 4 days (Fig. 9B), followed by scaffolds seeded with cells for 1 day. Acellular scaffolds had less basket-weave matrix than either of the cell-loaded scaffold samples, and all scaffolds elicited a better response than the sham wounds. In addition to a more normal matrix architecture, other features of wound healing appeared to be enhanced in defects implanted with fibroblast-seeded scaffolds. As shown in Fig. 10, scaffolds pre-seeded with fibroblasts appeared to stimulate greater regeneration of skin appendages including hair follicles than acellular scaffolds or sham wounds.

## Discussion

Engineered skin graft materials have evolved over time into more complex products with greater bioactivity; however, an ideal scaffold has yet to be developed. Current synthetic scaffolds, along with allograft or xenograft tissues, can elicit adverse clinical outcomes such as graft rejection, scar development, limited wound closure, bleeding, and infection [2]. There is an acute need for a skin substitute that can address these shortcomings. An optimal scaffold will incorporate biologic molecules that control cell function, appropriate mechanical properties, an architecture similar to native skin tissue, and a degradation rate that supports initial tissue formation, but does not hinder maturation of the regenerated tissue due to delayed resorption. [44]. Electrospinning is a technology that has existed for decades; however, it is increasingly being used in the tissue engineering field due to its simplicity, cost-effectiveness and capacity to produce nanofibrous scaffolds that mimic the structure of native skin tissue [45,46]. Moreover, synthetic and biologic molecules can be readily blended during the electrospinning process, allowing tuning of biochemical composition, mechanical strength and biodegradability. In this study we addressed one of the major limitations of electrospun scaffolds, the inherently small pore sizes, by mechanically introducing 160 μm diameter pores throughout the thickness of the scaffolds. The goal was to create spaces that would foster fibroblast infiltration into the scaffolds, followed by fibroblast-mediated secretion of native ECM. It is envisioned that for clinical translation, a commercial press containing 160 μm needles would be created, facilitating the production of scaffolds with highly reproducible pore sizes and spacing. Such presses are currently used in the cosmetics field for skin rejuvenation.

Scaffolds were electrospun using a 70% col I/30% PCL blend in order to generate a composite nanofibrous mesh that would support cell attachment, migration, and proliferation, and as reported previously [32], degrade with appropriate temporal kinetics. The scaffolds have a tensile modulus in the lower range of skin tissue [32], and as shown in this study, have sufficient tensile strength to withstand contractile forces applied by dermal fibroblasts. The scaffolds with seeded fibroblasts exhibited a contraction rate of 14 percent within the first 4 days, but did not contract any further over a 21-day interval. While one cannot directly compare *in vitro* and *in vivo* scaffold contraction, the capacity of the scaffolding material to constrain the amount of cell-induced contraction may be of benefit. Fibroblasts within granulation tissue differentiate into myofibroblasts, and then myofibroblasts direct wound contraction [47]. Some degree of wound contraction is necessary for proper healing, however excessive, or too rapid, contraction can hinder the formation of a normal dermal matrix architecture, causing scarring. Synthetic scaffolds may restrain contraction of the wound bed, allowing sufficient time for dermal regeneration [47].

To further enhance the regenerative potential of the 70:30 col/PCL scaffolds, 160 μm diameter micropores were created in order to promote fibroblast infiltration. Fibroblasts migrating into the micropores secreted native ECM molecules, including collagen I and fibronectin, resulting in a remodeled scaffold more similar to native dermal matrix. These results are in line with our prior investigation [32],which evaluated the response of human dermal fibroblasts to scaffolds with varying collagen/PCL ratios and pore sizes. In this prior study, 70:30 col/PCL scaffolds with 160 μm pores offered the best balance between suitable mechanical characteristics, biodegradability, and a cell-supportive biochemistry.

Recent reports have suggested that pre-seeding scaffolds with fibroblasts, keratinocytes, stem cells, or a combination of these has a beneficial role in wound healing [35,48–50]. The advantages that pre-seeded cells provide are thought to result from deposition of ECM proteins, as well as secreted factors such as cytokines and growth factors. Regenerated tissues within wounds implanted with cell-seeded scaffolds are reported to have a reduced concentration of dense collagen I, faster re-epithelialization, enhanced angiogenesis, and a higher degree of proliferative cells [50]. All of these factors have an influence on the wound healing rate, and also aid in reducing scar formation. In light of these findings, the objective of the current study was to determine whether pre-seeding 70:30 col/PCL microporous scaffolds with cells prior to implantation would enhance the wound healing response.

To test this hypothesis, acellular scaffolds, or scaffolds seeded with fibroblasts for either 1 or 4 days, were grafted into full-thickness critical size skin defects. It was found that both of the fibroblast-seeded scaffolds stimulated more effective wound healing than acellular scaffolds, and all of the scaffolds promoted better healing when compared with sham wounds. Moreover, scaffolds cultured with fibroblasts for 4 days elicited enhanced skin repair relative to scaffolds cultured with fibroblasts for only 1 day. Scaffolds pre-seeded with fibroblasts stimulated the formation of a dermal matrix with a basket weave-type architecture, similar to native unwounded skin. Additionally, cell-loaded scaffolds promoted markedly greater regeneration of skin appendages such as hair follicles. Both of these features were more pronounced in the scaffolds pre-seeded with fibroblasts for 4 days, as compared with 1 day. At present, the mechanism underlying the enhanced response to scaffolds cultured with cells for 4 days is unclear, however we hypothesize that the longer culture interval allowed greater deposition of ECM, as well as the possible accumulation of secreted cytokines and growth factors.

In conclusion, 70:30 col/PCL scaffolds with 160 μm pores support fibroblast survival, proliferation, and ECM deposition. Furthermore, the pre-seeding of these scaffolds with dermal fibroblasts prior to implantation stimulated the formation of more normal-appearing regenerated tissue within full-thickness skin defects.
