# Correction: Microporous Dermal-Mimetic Electrospun Scaffolds Pre-Seeded with Fibroblasts Promote Tissue Regeneration in Full-Thickness Skin Wounds

**DOI:** 10.1371/journal.pone.0126541

**Published:** 2015-04-15

**Authors:** The PLOS ONE Staff

The e-mail address for the corresponding author is incorrect. It should be: bellis@uab.edu.

The publisher apologizes for the error.
